# A narrative review of global inequities in access to uterine artery embolisation

**DOI:** 10.1186/s42155-026-00676-w

**Published:** 2026-03-28

**Authors:** Sara Lojo-Lendoiro, Greicy Heymann, Heather K Moriarty, Maja Wojno, Elika Kashef, Adam Plotnik, Rahil Kassamali, Andreas H. Mahnken, Vinicius AV Fornazari, Warren Clements

**Affiliations:** 1 Department of Interventional Radiology, Hospital Arquitecto Marcide, Ferrol, A Coruña, Spain; 2 Department of Radiology, Geneva University Hospitals (HUG), Geneva, Switzerland; 3 Faculty of Medicine, University of Bern, Bern, Switzerland; 4 Cork University Hospital, Cork, Ireland; 5 Interventional Radiology, Netcare Parklane Hospital, Johannesburg, South Africa; 6 Interventional Radiology, Department of Imaging, Imperial College NHS Trust, London, United Kingdom; 7 David Geffen School of Medicine at UCLA, Los Angeles, 90,095 United States; 8 Hamad Medical Corporation, Doha, Qatar; 9 Aston University, Birmingham, England; 10 Diagnostic and Interventional Radiology and Nuclear Medicine, St. Josef-Hospital, University Hospital, Ruhr University Bochum, Bochum, Germany; 11 Sector of Interventional Radiology and Angiography, Department of Diagnostic Imaging, Universidade Federal de São Paulo (EPM-Unifesp), São Paulo, SP Brazil; 12 Department of Radiology, Alfred Hospital, Melbourne, VIC 3004 Australia; 13 Department of Surgery, School of Translational Medicine, Monash University, Melbourne, VIC 3004 Australia; 14 National Trauma Research Institute, Melbourne, 3004 Australia

**Keywords:** Fibroid, Embolisation, Uterus, Global, Heavy menstrual bleeding

## Abstract

Uterine fibroids are the most common benign tumour of the female reproductive tract. For decades, surgery has been the standard treatment for symptomatic fibroids. However, minimally invasive options such as Uterine Artery Embolisation (UAE) have now emerged as a less invasive, but equally effective treatment. Despite this, access to UAE remains uneven worldwide. This cross-section from ten countries representing all inhabited continents across the globe, highlights several challenges that still exist, despite the abundance of evidence to support the long-term quality of life improvements for women treated with UAE. This narrative review considers enabling factors as well as barriers to treatment in each region, putting forward local strategies to improve access to UAE moving forward.

**Graphical Abstract:**

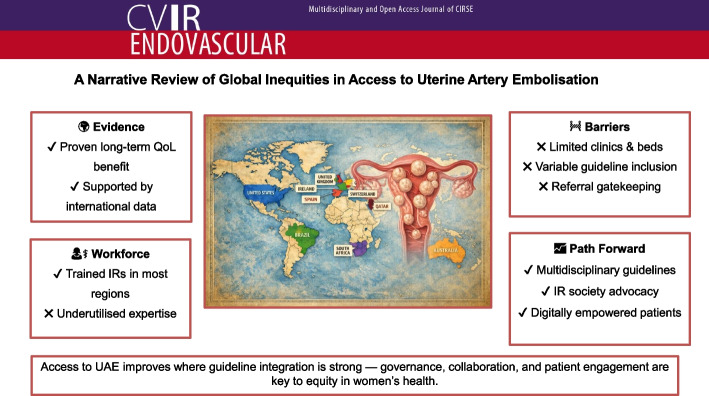

## Introduction

Uterine fibroids are the most common benign tumours of the female reproductive tract, with a prevalence estimated at 40–60% in women of reproductive age [1, 2]. The Global Burden of Disease 2019 study reported approximately 226 million prevalent cases and 9.6 million incident cases of uterine fibroids, with an increasing age-standardized incidence rate (average annual percent change 0.27%) and prevalence rate (0.078%) between 1990 and 2019 [3, 4].

Although many fibroids remain asymptomatic, 20–40% of women experience clinically significant symptoms such as heavy menstrual bleeding, pelvic pain, pressure on adjacent organs, or infertility, with a substantial impact on quality of life [2, 5]. Beyond physical health, fibroids exert a psychosocial burden, impairing mental well-being, sexual function, and social relationships [6].

For decades, hysterectomy has been the standard treatment for symptomatic fibroids, while myomectomy was reserved for women wishing to preserve fertility [6]. However, minimally invasive options such as Uterine Artery Embolisation (UAE) have now emerged as a less invasive, but equally effective treatment [7–9]. These are supported by level 1 evidence, and with quality of life outcomes up to 10 years post treatment [10, 11].

Despite this, access to UAE remains uneven worldwide. In this narrative review, the authorship consortium presents the current state of access to UAE in each of their own countries, presented alphabetically. This will summarise and contrast access and governance challenges in each region. This review will highlight geographic, socioeconomic, and racial disparities in its utilization, with surgery still predominating in all healthcare systems [12]. This creates a global inequality of opportunity, where not all women can equally benefit from a uterus-preserving therapy.

## Methods

A consortium authorship was formed from different countries that are represented in this review. Authors were given 5 different questions (Table 1) and asked to use these as a basis to contribute a short summary of the current state of access to UAE in their country, using local relevant evidence and opinion of the expert author and based on lived experience of practicing IR in their country. Completed responses were collated and are presented below, with a summary of responses presented in the discussion and in Table 2. As this was not a systematic review, it was not performed according to a standardised protocol, for example Preferred Reporting Items for Systematic reviews and Meta-Analyses (PRISMA).

**Table 1:** Questions used to stimulate a short summary of local access to UAE in each country

Questions	Example response
What is the current state of access to UAE*?	Qualitative response
What is the primary referral pathway?	Consider different approaches such as self-referral, primary car/general practice, gynaecologist, or other
Is UAE* integrated into local governance pathways?	Consider local societies, local government, Medicare,
What are local barriers to progression of access to UAE*?	Consider a range of evidence-based barriers that are relevant in your region
What are local factors that enable progress?	Consider a range of evidence-based barriers that are relevant in your region

^*^UAE uterine artery embolisation

**Table 2:** Summary of access and governance in the setting of treatment of symptomatic fibroids with uterine artery embolisation

	**What is the current state of access to UAE? Easy, difficult, no access**	**What is the primary referral pathway? Self, primary care, gynae, other**	**Is UAE integrated into local governance pathways?**	**What are the local barriers to progression of access?**	**What are local factors that enable progress?**
Australia	Available widely, but only in select metropolitan hospitals and thus under-utilised	Gynaecologists, self-advocating patients via GP	Yes, society guidelines and government pathways	Specialty access, awareness, negativity from gynaecologists	Social media, artificial intelligence
Brazil	Difficult as only available in tertiary centres and major cities	Self-referral driven by social media	Recognised by Ministry of Health but only incorporated into society guidelines	IR specialty recognition. Access to beds, reimbursement concerns, IR staffing	Society advocacy (SOBRICE), digital media
Germany	Widely available but under-utilised	Gynaecologists and GPs	No	Heavy gynaecologist oversight and lack of referral	Patient self-advocacy, society advocacy (DEGIR)
Ireland	Variable, mainly in tertiary centres	Gynaecologists	Yes	IR workforce shortage, lack of gynaecologist referrals, hospital bed pressure	Society and individual advocacy
Qatar	Available service and easy access—but limitations in referral pathway	Gynaecologists	No—international guidelines are used	Concerns about infertility. More awareness and education for patients and gynaecologists required	Closer communication with gynaecology and primary health care physicians
South Africa	Difficult	Self, gynaecologists and IR guided	Referral pathways are ill-defined	Lack of Access to advanced care, lack of awareness, socioeconomic factors	Social media, IR’s raising awareness
Spain	Difficult	Gynaecologists	Yes, society guidelines and government pathways	Unequal distribution of Interventional Radiology (IR) units. Lengthy referral pathways Restrictions in younger women. Logistical limitations in hospitals Limited visibility and recognition	Role of scientific societies. Close collaboration with gynaecology. Dissemination of scientific evidence Technical innovations
Switzerland	Difficult	Predominantly gynaecologists; rare self- or primary-care referrals	Recognition exists at the reimbursement level, but no structured clinical or governance integration is currently in place	No subspecialty recognition, limited outpatient infrastructure, referral variability, surgical culture, and ward-based logistical constraints	IR–gynaecology and IR–primary care collaboration, clinical advocates, social media engagement
United Kingdom	Heterogenous based on location (easy for teaching hospitals)	Predominantly gynaecologists	Yes, society guidelines, recommendations and government funded pathways	Lack of knowledge from patient and clinician, Lack of specialty IR status, therefore no direct pathway	MDT- IR attendance Speaking and building a rapport with gynaecologist IR led clinics for UAE work up
United States	Available, but underutilised	Mainly gynaecologists but also some self-referral	Yes, by several societies	Awareness, lack of IR services in regional/rural sites	Society advocacy, patient self-advocacy

### Australia

Unfortunately, access to UAE in Australia remains highly variable. Australia runs a National Health Service model with a predominantly public and a smaller private system [13]. All patients require triage through a general practitioner however both patients and general practitioners (GPs) suffer from a lack of awareness of not just interventional radiology (IR), but also UAE [14] leading to predominant gynaecologist referral. A previous study showed that surgery for fibroids was performed 41.8 times more often than embolisation in Australia [15]. This is despite UAE featuring appropriately as treatments for fibroids in both local IR [16] and gynaecology [17] guidelines, plus also featuring in government treatment pathways [18].

But lack of awareness and referrals aren’t the only issue. Many IRs don’t have direct access to essential services such as outpatient clinics and hospital admitting rights [19]. This is a direct result of the lack of government specialty recognition and the broad lack of recognition of IR with regulators and administrators, a decision which directly impacts patient access.

However, IRs are working hard towards change, and public recognition of IR and UAE through media and social media has been successful, albeit slow. In addition, patient-led healthcare decisions using objective artificial intelligence (AI) models that prioritise data over inherent biases will likely lead to better access in the future [20].

### Brazil

UAE is formally integrated into national governance frameworks in Brazil through the Brazilian Society of Interventional Radiology and Endovascular Surgery (SOBRICE), which explicitly endorses UAE in its official guidelines. As a result, UAE is recognized by the Brazilian Ministry of Health, leading to mandatory reimbursement by both private health insurance plans and the public health system [21]. Despite this regulatory incorporation, access to UAE across the country remains uneven.

Availability is largely concentrated in major metropolitan areas and private tertiary hospitals, where infrastructure, specialist distribution, and reimbursement models are more favourable. In many mid-sized cities and rural regions, UAE remains limited or unavailable. This geographic disparity reflects systemic imbalances in resource allocation, health insurance penetration, and human-resource distribution [22].

Although self-referral has increased due to social-media driven patient awareness and communication initiatives led by SOBRICE [23, 24], overall utilization remains below expectations. Low physician and patient awareness, along with variable acceptance of UAE among gynaecologists and health insurers, continues to hinder broader adoption which maintains a gatekeeping role in fibroid-related care.

Key national barriers include the incomplete recognition of IR as an independent specialty, limited availability of inpatient beds, reimbursement concerns, and insufficient IR staffing.

Conversely, enabling factors include strong national advocacy from SOBRICE, expanding patient-led demand, the growing scientific literature supporting UAE, and the impact of digital platforms in disseminating evidence and demonstrating clinically meaningful improvements and reproductive potential preservation [25–27]. Collectively, these forces have contributed to gradual yet measurable expansion of UAE access throughout Brazil.

### Germany

The role of UAE in treating symptomatic uterine fibroids is still under discussion in Germany, but treatment is widely available. There are some regional variations depending on the number of hospitals offering IR services. However, an analysis of the quality management system of the German Interventional Radiological Society (DEGIR) proved the widespread availability of embolisation services for a variety of medical conditions [28, 29].

Both general practitioners and gynaecologists can refer patients for UAE. In addition, a limited number of patients self-refer. According to an interdisciplinary consensus between IR and gynaecology dating back to 2019, any patient considered for UAE should be examined by a gynaecologist prior to treatment [30].

UAE is not currently recommended as a primary treatment in national guidelines. A national guideline on the treatment of benign uterine conditions, including fibroids, was initiated in 2020 [31]. However, this guideline is not yet available.

Despite scientific evidence supporting UAE as an effective treatment for uterine fibroids and sufficient reimbursement, referral numbers throughout Germany remain relatively low, with surgical treatment continuing to be prioritised over UAE. To facilitate the use of UAE in Germany, DEGIR is advocating for the inclusion of fibroid treatment in the Federal Joint Committee's (G-BA) second opinion policy [32].

### Ireland

UAE is an established treatment option for fibroid-related heavy menstrual bleeding in Ireland. It is included in Health Service Executive (HSE) clinical guidance for the investigation and management of menorrhagia [33]. International standards e.g. Cardiovascular and Interventional Radiology Society of Europe (CIRSE) are also used to inform local practice.

Access in Ireland is variable: UAE is available in several tertiary centres (public and private), but service distribution is uneven and true nationwide access can be difficult outside major hospitals. The common referral pathway is via gynaecology services (referral from GP to hospital gynaecology clinic, then onward referral to interventional radiology). Referrals directly from primary-care and self-referral pathways are uncommon. If referred in this format, IRs typically involve a gynaecologist in the patients care prior to proceeding with UAE. Patients are typically seen for pre- and post-procedural outpatient care by the interventional radiologist in outpatient clinic.

Key local barriers include workforce and service capacity (shortages of interventional radiologists), variable local gynaecology–IR care pathways (driven by concerns of procedural complications) and hospital resource/bed pressures [34].

Enablers of UAE are active local IR and gynaecology advocates. Published Irish research and case series, tertiary centre leadership, professional society guidance and patient information resources also raise awareness and support pathway development for routine UAE.

### Qatar

In Qatar, interventional radiology is mainly delivered through the government healthcare system, with limited provision in the private sector. UAE is available within public hospitals, however the number of procedures performed remains low relative to the size and diversity of the female population. This raises an important question: why is UAE underutilised?

Culturally, there is a strong emphasis on having large families, and preserving fertility is often a central priority even in older age groups. As there are no Qatar-specific fibroid management guidelines, gynaecologists generally follow international guidance, which traditionally recommends myomectomy as the preferred option for women wishing to conceive [35]. Yet many of these recommendations are based on older evidence and do not reflect the growing data supporting UAE as a safe, uterus-preserving option in selected women [36]. Consequently, referral rates to interventional radiology in Qatar remain low.

Recently, IRs have seen an increase in patient-driven referrals, particularly from women who have researched treatment alternatives and are seeking minimally invasive options. To support informed choice, greater public education around UAE is needed. There are now established dedicated interventional radiology clinics for counselling and shared decision-making, and IRs regularly engage with gynaecology and primary care colleagues to ensure that women in Qatar are offered the full spectrum of evidence-based fibroid treatments.

### South Africa

Despite the significant disease burden of uterine fibroids in Sub-Saharan Africa (SSA), the UAE procedure is not readily available to the vast majority of these women due to limited access to advanced care and IR in the region [37]. Challenges include corrosive poverty, transportation limitations, wrongful, deep-seated cultural beliefs, where fibroids are often seen as a demonic curse, and a lack of education. This is confounded by limited access to appropriate imaging, high costs, and poorly-trained personnel, particularly in remote regions [38, 39]. In addition, most SSA women do not carry health insurance to cover the expense of fibroid disease management [39].

Although UAE is mentioned in the clinical guidelines by the South African Society of Obstetricians and Gynaecologists (SASOG) as a treatment option for fibroids, it is notably not included as an option for adenomyosis, for which a hysterectomy is recommended if medical treatment fails [40].

A South African study noted that many of its participants reported very limited information from healthcare clinicians about fibroids, and most participants obtained information about the disease from the internet, friends, or other patients [38].

The onus has therefore primarily been on IR specialists to raise awareness, not only to their patients but also to colleagues, for UAE as a well-established treatment option for these conditions. Additionally, referral pathways remain poorly defined. UAE is therefore still a far-reaching target as a treatment option for patients in SSA, with much education, awareness, and research to be done to increase its access in our continent.

### Spain

UAE is available in Spain, mainly in tertiary hospitals belonging to the National Health System (SNS) but also in private centres. However, there are regional inequalities between autonomous communities, as not all hospitals offer IR services.

Referrals to UAE are usually made through hospital gynaecology departments, which, after clinical evaluation, propose embolisation as an alternative to surgery. Although primary care is the point of entry to the system, in practice a gynaecological evaluation is required prior to referral.

UAE is integrated into national governance and included in the SNS portfolio under diagnostic and therapeutic IR. There are regional guidelines [41] and regional service portfolios [42] that include UAE as an option for symptomatic fibroids, with caution in women who wish to become pregnant. National protocols follow the recommendations of the Spanish Society of Vascular and Interventional Radiology (SERVEI), which has published patient information and technical updates on embolisation [43, 44].

Despite strong evidence supporting UAE as a safe and effective technique [45, 46], several barriers remain: limited distribution of IR units, long referral pathways, logistical requirements such as beds and anaesthetic support, and reluctance to indicate UAE in young women despite newer evidence [47–49]. In clinical practice, surgical options continue to be prioritised in this population as a precautionary measure.

Facilitating factors include the role of SERVEI, close collaboration with gynaecology, the dissemination of scientific evidence, and technical advances such as transradial access and improved perioperative and postoperative analgesia, which improve both clinical outcomes and patient acceptance.

### Switzerland

Access to UAE in Switzerland remains heterogeneous and largely dependent on local institutional structures. The procedure is available in all major university hospitals as well as in several non-university and private centres, yet nationwide access remains inconsistent.

Most referrals originate from gynaecologists, who traditionally manage fibroid-related symptoms and often remain the gatekeepers of therapy. Direct patient self-referral is uncommon, as awareness among primary care physicians and among patients themselves regarding UAE remains limited. Many women undergoing hysterectomy or myomectomy are not informed that embolisation is a valid therapeutic option [50].

At a government level, UAE is recognised within the Swiss diagnostic reference group (DRG) reimbursement catalogue [51], and IR acts are officially coded under frameworks regulated by the Federal Office of Public Health (BAG) [52]. However, there are no national clinical guidelines specifically integrating UAE into gynaecological care pathways, and no national registry exists to track UAE procedures or outcomes, making it difficult to quantify activity and monitor quality indicators. Institutional protocols vary widely, reflecting Switzerland’s decentralised healthcare system and the absence of formal multidisciplinary fibroid boards. In some hospitals, IRs are not included in fibroid or pelvic imaging boards, which are often led by diagnostic radiologists or gynaecologists.

Barriers to broader access include the absence of an officially recognised subspecialty status for IR in Switzerland, limited availability of outpatient consultation infrastructure, and variability in referral practices among gynaecologists. In most hospitals, post-procedural care is provided within gynaecology wards, where bed availability may constrain procedural scheduling and overall throughput. Facilitating factors include strong local champions in academic, non-university, and private sectors, collaborative IR and gynaecology initiatives, and advocacy through the Swiss Society of Vascular and Interventional Radiology (SSVIR), which actively disseminates patient information and promotes awareness of minimally invasive fibroid treatment options [53].

### United Kingdom

Access for UAE, as with most women’s health treatments, remains variable in the United Kingdom (UK) [54]. UAE is a recognised procedure by the National Institute for Health and Care Excellence (NICE) who have recommended UAE since 2007 due to its established safety and efficacy profile. This led to UAE being supported and funded by the National Health Service (NHS). Furthermore, The Royal Colleges of Obstetricians and Gynaecologists (RCOG) and Radiologists (RCR) have published joint clinical recommendations recommending UAE in the management of fibroids [55]. Despite this, a recent study showed the Myomectomy/UAE ratio remains 60%/40% in the UK [56].

The primary referral pathway is predominantly from gynaecology, followed by multidisciplinary review, attended by an IR. Self-referral or direct GP referral to IR are not commonplace due to the nature of the NHS funding pathways.

Obstacles for equitable access remains awareness, both at a patient and gynaecologist level. As with other countries, concerns have been raised that lack of specialty status for IR is a significant barrier for UAE referrals from primary care.

The British Society of Interventional Radiology (BSIR) have been offering patient information leaflets online [57] and advocating for role of IR in fibroid management. With more publications being open-access and use of AI search tools, as well as patients taking ownership of their health, and patients results/Reports being available to patients almost as soon as the clinician, UAE are coming into the limelight more and will no doubt will increase in presence and activity across the UK.

### United States of America

In the United States (US), access to UAE is relatively available but underutilised. A recent study showed that although UAE procedures have increased over the years, it still lags behind surgery which accounts for approximately 95% of fibroid interventions [58]. Patients older than 40 years, African-American, those enrolled in Medicaid/Medicare, and those living in lower-income communities were more likely to undergo UAE. Whereas patients at rural hospitals, white patients, and those with private insurance were more likely to undergo surgical interventions.

UAE is integrated into clinical guidelines and coverage frameworks in the US. The American College of Radiology (ACR) updated its Appropriateness Criteria in 2023 which include UAE as a treatment option [59]. The Society of Interventional Radiology (SIR) offers advocacy and coverage guidance for UAE, as well quality improvement guidelines [60] and access to vetted patient information including videos of lived experiences [61]. The American College of Obstetricians and Gynecologists also lists UAE is an option for symptomatic uterine fibroid, however considers it “relatively contraindicated” for women who strongly desire future fertility [62]. As a result, Medicare/insurance coverage is generally supportive when criteria are met, though approvals vary [63].

Referral pathways in the US typically involve a patient being seen by their primary care physician or gynaecologist and then being referred to an IR for evaluation. A small proportion of patients present as self-referrals which has led to direct marketing campaigns for awareness of the procedure [64]. Local barriers hindering wider access include relative lack of awareness among gynaecologists about UAE as an option [58], lack of IR programs especially in rural or less-resourced hospitals and providers perceptions that UAE is less established.

## Discussion

As summarised in Table 2, this cross-section from all inhabited continents across the globe representing the residing country of the authorship consortium, highlights several common challenges that still exist despite the abundance of evidence to support the long-term quality of life improvements for women treated with UAE.

In highlighting common themes, almost all countries report a workforce of IRs that are trained and able to contribute to the management of symptomatic fibroids. However, several countries report poor access to clinics and beds, with the lack of specialty recognition contributing to underutilisation of what IR can offer patients.

In comparing integration into local guidelines, marked variation was observed. Some regions such as the UK and Australia show integration into both government and society documents, while other regions have no guidelines and rely entirely on broader international guidance from organisations such as CIRSE. No regions report access on par with surgical alternatives performed by gynaecologists, however the greatest access correlated with locations having heavy guideline integration and as such, the authors stress the value of overarching evidence-based governance processes which have immense value in improving patient access.

Another common theme in each country was that gynaecologists still provide a gatekeeping role to patient access, with oversight of referrals, and this is combined with a relative lack of gynaecologist awareness of the outcomes of UAE based on contemporary evidence. This has led to many patients self-referring (where the healthcare system allows) or seeking a referral through self-advocacy with their primary care provider.

As a select group of countries representing the authorship’s lived experiences, this does limit the content of this review despite it covering a diverse range of regions. In addition, while the review was structured around the headings in Tables 1 and 2, it was not completed to the PRISMA guidelines. This means the reporting from different countries was not formally standardised nor systematic.

## Conclusion

Looking forward, it is abundantly clear that more work is needed to ensure that patient access can improve. In all regions, local eminent IR societies are playing a key role in advocating for better awareness, education, and sharing resources. However, these societies must continue to ensure governance systems reflects evidence including forming multidisciplinary guidelines with the highest reputational standing. This strategy must be combined with recognising the modern patient who is digitally-aware, social media ready, and using artificial intelligence tools. In these places, non-evidence-based biases are less pronounced. It is these activities that should be harnessed to drive change, improve equity, improve access, and help ensure that IRs contribute to shared informed decision making in women’s global health.

## Data Availability

N/a.
