# Correction: A narrative review of global inequities in access to uterine artery embolisation

**DOI:** 10.1186/s42155-026-00738-z

**Published:** 2026-07-15

**Authors:** Sara Lojo-Lendoiro, Greicy Heymann, Heather K. Moriarty, Maja Wojno, Elika Kashef, Adam Plotnik, Rahil Kassamali, Andreas H. Mahnken, Vinicius A. V. Fornazari, Warren Clements

**Affiliations:** 1 Department of Interventional Radiology, Hospital Arquitecto Marcide, Ferrol, A Coruña Spain; 2 Department of Radiology, Geneva University Hospitals (HUG), Geneva, Switzerland; 3 Faculty of Medicine, University of Bern, Bern, Switzerland; 4 Cork University Hospital, Cork, Ireland; 5 Interventional Radiology, Netcare Parklane Hospital, Johannesburg, South Africa; 6 Interventional Radiology, Department of Imaging, Imperial College NHS Trust, London, United Kingdom; 7 David Geffen School of Medicine at UCLA, Los Angeles, 90,095 USA; 8 Hamad Medical Corporation, Doha, Qatar; 9 Aston University, Birmingham, England; 10 Diagnostic and Interventional Radiology and Nuclear Medicine, St. Josef-Hospital, University Hospital, Ruhr University Bochum, Bochum, Germany; 11 Sector of Interventional Radiology and Angiography, Department of Diagnostic Imaging, Universidade Federal de São Paulo (EPMUnifesp), São Paulo, SP Brazil; 12 Department of Radiology, Alfred Hospital, Melbourne, VIC 3004 Australia; 13 Department of Surgery, School of Translational Medicine, Monash University, Melbourne, VIC 3004 Australia; 14 National Trauma Research Institute, Melbourne, 3004 Australia

**Correction: CVIR Endovasc 9, 34 (2026)**

**https://doi.org/10.1186/s42155-026–00676-w**

Following the publication of the original article [1], the authors reported the article category should have been “Review Article” but is incorrectly written as “Editorial” in the final version.
